# Hairy Cell Leukemia: A Differential Diagnosis of Hepatitis B-Associated Aplastic Anemia and Syphilis

**DOI:** 10.3390/hematolrep17020013

**Published:** 2025-03-15

**Authors:** I. Kindekov, E. Beleva, M. Kadish, I. Ionchev, N. Semerdzhieva

**Affiliations:** 1 Clinic of Hematology, Military Hospital, 1606 Sofia, Bulgaria; ivankindekov@gmail.com (I.K.); elina.beleva@biomed.bas.bg (E.B.); 2 QSAR and Molecular Modelling, Institute of Biophysics and Biomedical Engineering, Bulgarian Academy of Sciences, 1784 Sofia, Bulgaria; 3 Clinic of Internal Medicine, Hepatology and Gastroenterology, University Hospital ‘SOFIAMED’, 1797 Sofia, Bulgaria; medain@abv.bg; 4 Clinic of Internal Medicine, University Hospital of Emergency Medicine ‘Pirogov’, 1606 Sofia, Bulgaria

**Keywords:** hairy cell leukemia, acquired aplastic anemia, hepatitis B, syphilis

## Abstract

Aplastic anemia occurs with an incidence of 2–5: 1 million people worldwide. However, the frequency of newly diagnosed cases of bone marrow aplasia is greater, and some of these patients present to emergency departments initially. **Description of Case:** We present the case of a middle-aged man with pancytopenia. In this case, aplastic anemia associated with hepatitis B and syphilis was only the initial diagnosis. An indolent hematologic malignancy—hairy cell leukemia—was diagnosed as the real cause of the bone marrow failure in a clinic of hematology. **Conclusions:** This clinical case allows us to make a conclusion, albeit not definitively, about the contribution of hepatitis B and syphilis to the clinical manifestation of hairy cell leukemia. A detailed and consistent diagnostic plan is also required in patients presenting with pancytopenia. Failure to diagnose a hepatitis B infection in a patient with malignant hematologic disease would lead to fatal therapeutic errors.

## 1. Introduction

Aplastic anemia has an incidence of 2–5 per 1 million people worldwide [1]. However, the frequency of newly diagnosed cases of bone marrow aplasia is higher. Hepatitis-associated aplastic anemia (HAAA) is an uncommon variant of aplastic anemia in which pancytopenia occurs two to three months after acute hepatitis [2]. Several hepatitis viruses, including the hepatitis B virus (HBV), are associated with HAAA. Genetic factors, including mutations in the telomere repair gene complex that reduce the bone marrow’s ability to repair, are suggested as causes of this disorder [2]. Some of these patients initially present to emergency departments and internal medicine clinics.

Hairy cell leukemia (HCL) is a rare chronic B-cell lymphoid malignancy with a mean age at diagnosis of 55 years and an unexplained male predominance of affected patients. A clonal population of leukemic cells (mature activated memory B cells with usually hypermutated immunoglobulin genes) predominantly infiltrate the marrow, the spleen, and the liver in HCL. The most common presenting symptoms are weakness and fatigue due to anemia (50%), symptoms of frequent infections (17% active infections at presentation), and bleeding manifestations (severe thrombocytopenia). These are related to pancytopenia, which results from impaired hematopoiesis with various mechanisms of origin. Also, many patients with late detection of the disease report fullness in the left abdomen, early satiety, and discomfort with gradual onset associated with an enlarged spleen (advanced disease). Diverse autoimmune findings in patients, such as migratory inflammatory joint pain and edema, vasculitic skin lesions, and erythema nodosum, represent unusual complications.

## 2. Case Description

We consider it important to raise awareness of the association of hepatitis B-associated aplastic anemia with hairy cell leukemia (HCL) through the case of a 47-year-old man. He came to our hospital with complaints of extreme fatigue, shortness of breath and palpitations on exertion, leg–hand–face edema, dizziness, abdominal discomfort, nausea, and anorexia. The physical examination revealed pale, cold skin and conjunctivae. No apparent changes in nail beds and tongue except dry skin and mucous linings were detected. There was diffuse skin and subcutaneous tissue edema. No purpura, ecchymoses, or other types of skin rashes were visible. Mildly enlarged nontender palpable lymph nodes in the neck area were detected unilaterally, consistent with a history of recent jaw pain and presumed untreated dental infection. The percussion of lung fields showed no pleural effusions or infiltrates. No pathologic sounds were heard at lung auscultation. The borders of the heart showed no signs suggestive of cardiomegaly. The apex cordis was not visible or palpable. There were no visible pulsations of carotid arteries or vascular murmurs. His blood pressure was 110/70 mmHg, and his heart rate was regular but a bit tachycardic at 104/bpm at rest. The heart sounds were loud; no pathologic sounds or murmurs could be heard at auscultation in either the upright or left lateral decubitus positions. The palpation of the abdomen did not evoke pain, but the liver border was 4 cm below the costal margin, and the spleen was moderately enlarged. These symptoms developed gradually over the last 5 months. The patient had no history of chronic diseases or exposure to myelotoxic drugs and denied the use of myelotoxic substances at his workplace. He reported being previously treated for a syphilis infection more than 10 years ago. The laboratory test results showed pancytopenia.

## 3. Results

The laboratory tests are presented in Table 1.

In addition to neutropenia, immunologic tests revealed low numbers and percentages of natural killer (NK) cells, as well as high levels of IgE, and no evidence of autoimmune disease (extended tests for anti-nuclear antibodies, anti-liver antibodies, antithyroid antibodies, and anti-erythrocyte antibodies were negative).

**Figure 1:**
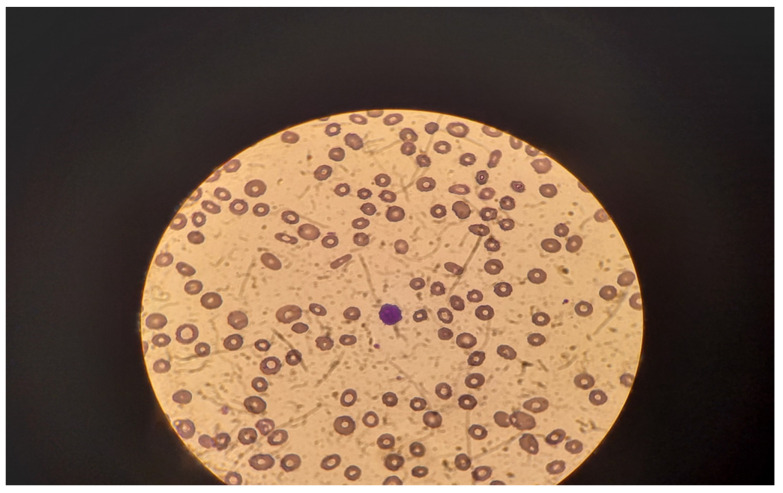
Peripheral blood cell smear—atypical lymphoid cell with ciliated cytoplasm—in the center.

An enlarged liver (191 mm along the medial clavicular line) and spleen (200/80 mm) were detected at the abdominal ultrasound. A pathologic portal blood flow was the main finding on Doppler echocardiography. There were no signs of free abdominal fluid or pleural effusions. A peripheral blood smear performed in our clinic revealed anisocytosis, poikilocytosis, and single oxyphilic erythroblasts. In the background of extreme lymphocytopenia, a single atypical lymphoid cell with ciliated cytoplasm was found per field (Figure 1).

The results of the assigned serological tests on the second day of the initial hospitalization in the clinic of internal medicine were positive for hepatitis B surface antigen, and the treponemal test was positive (HIV 1/2 and hepatitis C were excluded). A packed red blood cell transfusion was administered, and treatment with a third-generation parenteral cephalosporin was initiated upon admission. A high titer of specific anti-syphilis TPHA antibodies (1:320) was detected by the second test (using Western blot analysis) at the end of antibiotic therapy.

The patient was referred to a specialized clinic of hepatology and gastroenterology, where advanced virologic testing was performed. Despite normal transaminases, the patient had evidence of active viral replication (Table 2). Coinfections with HIV 1/2 and other hepatitis viruses were excluded (Table 2).

Gastroscopy revealed no evidence of esophageal varices or portal gastropathy. A liver biopsy was not performed due to thrombocytopenia and impaired coagulation. Therapy was started with the nucleoside analog (NUC) tenofovir disoproxil fumarate (TDF) 245 mg once daily. Flow cytometry of a peripheral blood sample performed at the hematology clinic showed 6% polyclonal B lymphocytes and 2.4% monoclonal B cells with antigen characteristics of hairy cell leukemia: CD19+/CD5−/CD103+/CD123+/CD11c+/CD25+/lambda+. The bone marrow biopsy showed hypercellular bone marrow and interstitial lymphoid infiltrates of small lymphocytes, with dense chromatin and pale cytoplasm, creating a cellular appearance. The bone marrow parenchyma was represented by sparse islands amid the lymphoid infiltrates. The megakaryocytes had a single nucleus, and hypolobulated megakaryocytes were the predominant type. The specimen exhibited erythroid lineage proliferation and low-grade maturation of red cells. The myeloid lineage was represented only sparingly. The reticular network was moderately enlarged (Gomori staining). The immunohistochemical study revealed that 90% of the CD20+ lymphoid cells were CD25+; Cyclin D1+ cells were positive in a small percentage of the leukemia cells; CD1a, TdT, and CD10 were not expressed by the bone marrow cells; and CD34+ cells were present in 1–2% of the blasts. The reticulocyte count was assessed after transfusion of reduced—41 × 10^9^/L, a borderline value characterizing patients with mild aplastic anemia. Cladribine (0.14 mg/kg) and dexamethasone (16 mg) were administered according to the 5-day regimen. One month after therapy with cladribine, the results still did not indicate a hematologic response. The enlargement of the spleen and liver persisted at repeated abdominal ultrasounds. The patient received packed red blood and platelet transfusions during this follow-up hospital admission. A bone marrow reassessment was scheduled 4–6 months after the treatment. We have no results of follow-up plasma HBV Ag, HBV Ab, and HBV DNA copies of the patient after the therapy with cladribine. A repeated admission in the clinic of gastroenterology and hepatology was recommended to the patient for control tests one month after the chemotherapy. However, he has not complied with this recommendation. We believe that he had taken tenofovir regularly for more than one month after chemotherapy because he had an issued prescription for tenofovir entirely pre-paid by the National Health Insurance Company. The patient was lost for effective follow-up.

## 4. Discussion

Syphilis and viral hepatitis B remain among the differential diagnoses for anemia and thrombocytopenia when other, more common causes have already been excluded [3,4]. Various immune disorders have been reported in HAAA, including lymphocytopenia, hypogammaglobulinemia [5], imbalance of the T-cell immune response, a low CD8/T-cell ratio [6], an increased number of cytotoxic cells [7], and neutropenia [8]. Bacterial, fungal, or other infections may occur as a second infection in a patient presenting with HAAA [9]. Early-stage T. pallidum infection also involves antigen-specific proliferation of cells in the spleen and lymph nodes days after infection, with persistent proliferation lasting for many weeks to months [10]. This is accompanied by a slight decrease in the total percentage of CD4+ T cells and a proportional increase in CD8+ T cells [10,11,12]. The peripheral blood results revealed a low number and percentage of NK cells, high IgE levels, and neutropenia in our patient. Hematological manifestations of syphilis include anemia and thrombocytopenia [13,14,15,16] and can be explained by several causes, including abnormal hematopoiesis [14]. In the past, several types of anemia were distinguished in adults with syphilis: anemia associated with the inflammatory process, anemia in advanced leukemia, a very rare form of pernicious anemia, and anemia as a side effect of treatment with chlorine-containing preparations in the pre-antibiotic era [13]. To date, T. pallidum has been reported as a possible cause of pancytopenia only in diseased adults with hepatitis C or HIV co-infection [3,5]. Hemolytic anemia and thrombocytopenia have been described before in congenital syphilis. Anemia in congenital syphilis resembles that of fetal erythroblastosis [16] or is interpreted in the context of hemophagocytosis [17].

Idiopathic aplastic anemia shares common pathogenetic aspects with a rare autoimmune hemolytic anemia in adults called paroxysmal nocturnal hemoglobinuria (PNH) [18]. Historically, PNH was almost invariably observed as a chronic or relapsing condition in patients with late diagnosis of syphilis that has remained untreated. Without specific evaluation for PCH, the condition may be misdiagnosed [19]. PNH mutant cells seem to be present in a majority of patients with aplastic anemia initially, and a significant percent of these patients with aplastic anemia (and PNH) mutant cells could evolve to a typical hemolytic PNH [18]. The presence of PNH is related to immunologically mediated marrow failure and is a positive predictor of the response to immunosuppressive therapy in aplastic anemia [20]. PNH is characterized by an unusual “biphasic” autoantibody that binds to red blood cells (RBCs) in cold temperatures or cooler areas of the body. Upon rewarming, a complex cascade involving the complement system ultimately induces cellular destruction. The tests for erythrocyte agglutination at low and high temperatures separately were negative, so there were no detectable anti-erythrocyte antibodies (a similarity with the presented patient). On the other hand, though lactate dehydrogenase was elevated and haptoglobin was not measured, other tests demonstrating hemolysis (total bilirubin, reticulocytes) were not indicative of PNH in the case of our patient. He did not report symptoms suggestive of episodes of hemoglobinuria.

Unfortunately, the stage of syphilis infection of this patient remained unspecified according to our data. The serological response to syphilis treatment is evident as early as 6 months in early syphilis patients [21]. Only 17.1% of patients who had an adequate response to syphilis treatment achieved complete reagin (RPR) seroconversion 12 months after therapy. Baseline reagin titers ≤ 1:32 are associated with increased odds of seroconversion [22]. Notably, the results of specific treponemal tests, such as the T. pallidum hemagglutination test (TPHA), are positive in only 11.3% of patients with false-positive reagin (nontreponemal) tests for syphilis [23].

Antiviral treatment is prescribed for HBeAg-negative hepatitis patients with viral DNA levels >10,000 copies/mL and either a twofold increase in ALT and/or histologic activity (>A2) or fibrosis (>F2) [24,25]. When immunosuppressive treatment or chemotherapy is necessary, antiviral prophylaxis with NUC is recommended for any patient with a positive HBsAg or high anti-HBc total without a high anti-HBsAg titer, regardless of viremia, to prevent viral reactivation [24,25]. Pancytopenia in the course of hepatitis B necessitates a work-up to diagnose late complications of the infection, such as myelodysplastic syndrome [26], lymphoma [27], or other malignant lymphoproliferative diseases. The rapid improvement in hematopoiesis in patients with isolated hepatitis-associated aplastic anemia was primarily associated with a reduction in the viral load reported after one month of TDF (Tenofovir) treatment [2]. The anemia of the patient could be co-incidental and not related to hepatitis B. We were unable to confirm any hypothesis due to an obscure sequence of patient’s diseases in the past. Pancytopenia (including hyporegenatory anemia) is characteristic for hairy cell leukemia (HCL). Hairy cell leukemia (HCL) is an uncommon B-cell lymphoproliferative disorder with a prevalence of 2% of adults with leukemia and was first reported as a distinct disease in 1958 [28]. Anemia in HCL may result either from extensive marrow infiltration with leukemic cells or from fibrosis of bone marrow induced by the overproduction of cytokines by the leukemic cells or from impaired hematopoiesis related to inadequate growth factor production. Autoimmune hemolytic anemia is also documented in patients with HCL. This autoimmune phenomenon is not related to tumor burden, and it may be present at initial diagnosis or occur at any time throughout the course of the disease. Also of note, hypersplenism and portal hypertension could cause the counts of granulocytes and platelets to go down.

In this case, hepatitis B-associated aplastic anemia was the initial diagnosis. We found reports, mostly clinical cases, showing the association of hepatitis B with the development of hairy cell leukemia, although a mechanism underlying the association of these two diseases has not been established [29]. The strongest epidemiological correlations the carcinogenic capacity of HBV were with the occurrence of B subtypes non-Hodgkin’s lymphoma (Svicher). HBV DNA integration is recognized as a mechanism promoting neoplastic transformation by several mechanisms, including the direct dysregulation of oncogenes and onco-suppressors, the production of chimeric viral-human RNAs and proteins with transactivating properties, and the promotion of overall genome instability. The presence of HBV DNA in peripheral blood mononuclear cells has been reported by numerous studies. HBV DNA integration in the exonic regions of some genes and also in coding genes was confirmed and was viewed as potentially involved in mechanisms underlying carcinogenesis. It is also possible that HBV acts more as an inflammatory driver than as a direct carcinogen at the onset of a hematological malignancy. Some in vitro studies have shown the ability of HBV to infect both lymphomonocytes and bone marrow stem cells, whose differentiation is halted by the virus. The demonstrated persistence of HBV DNA in peripheral lymphomonocytes and bone marrow stem cells suggests that these cellular compartments may act as HBV reservoirs, allowing viral replication to resume later in the immunocompromised patients [30]. Currently, the association of chronic hepatitis B infection with the development of hairy cell leukemia, in particular, is not supported by studies [1,31].

Finally, the purine nucleoside analogs that are used as a backbone of induction therapy can produce profound and prolonged myelosuppression. Because of the extensive marrow involvement with leukemia, the myeloid reserve is severely compromised at the initiation of therapy. Following effective therapy, the granulocytes gradually recover, but the purine nucleoside analogs usually induce a prolonged period of reduction in lymphoid cells and risk of opportunistic infections (and infections overall) resulting from compromised lymphocyte function. The risk is higher with a chemotherapy regime including cladribine and rituximab (CD20 monoclonal antibody against B-lymphocytes) compared to monotherapy (e.g., cladribine). Once the patient has achieved complete remission, the risk for infection becomes progressively less as the hematologic parameters improve.

## 5. Conclusions

The presented clinical case allows us to draw conclusions, albeit not definitively, about the contributions of hepatitis B and syphilis to the clinical manifestations of hairy cell leukemia. Failure to diagnose a hepatitis B infection in patients with malignant hematologic disease can lead to life-threatening therapeutic errors.

## Figures and Tables

**Table 1:** Results of the laboratory test.

Test	Baseline Result	Follow Up Result	Reference Range
WBC × 10^9^/L ^†^	0.7	1.52	4.1–11.0
Lymphocytes × 10^9^/L	0.6 (74.5%)	1.41 (92.8%)	0.6–4.1 (20–40%)
Granulocytes × 10^9^/L	0.1 (20.4%)	0.08 (5%)	2.0–7.8 (54–76%)
Monocytes × 10^9^/L	0.01 (1.2%)	0.02 (1.5%)	0.10–1.0 (2–10%)
Eosinophils × 10^9^/L	0.01 (1.0%)	0.01 (0.5%)	0.0–0.6 (0–6%)
Basophils × 10^9^/L	0.0 (0.1%)	0.0 (0.2%)	0.0–0.1 (0–1%)
MID cells × 10^9^/L	0.0 (5.1%)	0.0 (0.1%)	0.0–0.1 (0–1%)
Erythrocytes × 10^12^/L	1.18	1.92	4.6–6.3
Hemoglobin, g/L	39	66	140–180
Hematocrit, l/L	0.13	0.21	0.40–0.51
MCV fl ^╣^	109	110	82–98
MCH pg ^╧^	33.1	34.6	26.0–32.0
MCHC g/L ^¥^	304	314	300–360
RWD% ^≠^	21.3	26.3	11.5–14.5
Platelets × 10^9^/L	30	18	140–440
Sedimentation rate mm	-	28	0–15
Glucose, mmol/L	8.15	4.2	4.1–5.9
Urea, mmol/L	4.7	4.2	2.8–7.2
Creatinine, µmol/L	82.0	73.0	74–110
Albumin, g/L	32	38	36–52
Protein total, g/L	75	63	66–83
Bilirubin total, µmol/L	16.0	18.8	5.0–21.0
Bilirubin direct, µmol/L	4.5	4.4	0.0–3.4
C-reactive protein, mg/L	14.0	0.14	0.0–0.50
ASAT U/L ^‡^	12	7	0–50
ALAT U/L ^#^	12	10	0–50
GGT U/L ^§^	21	25	0–55
α-Amylase, U/L	34	50	28–100
Alkaline phosphatase, U/L	39	33	30–120
Lipase, U/L	12.0	15.5	0–67
Potassium, mmol/L	3.9	4.0	3.5–5.1
Sodium, mmol/L	136	138	136–146
Chloride, mmol/L	104	105	98–111
Lactatdehydrogenase, U/L	86	-	0–248
Ferritin, ng/mL	442.6	-	20–250
aPTT, s ^₽^	-	29.5	26.4–36.8
Fibrinogen, g/L	5.0	3.0	1.5–4.5
Prothrombin time, s	16.9	13.1	8.0–13.2
INR ^Ω^	1.55	1.18	0.8–1.2

Legend: ^†^ WBCs—white blood cells; ^╣^ MCV, fl—mean corpuscular volume, femtoliter; ^╧^ MCH—mean corpuscular hemoglobin; ^¥^ MCHC—mean corpuscular hemoglobin concentration; ^≠^ RWD—red cell distribution width; ^‡^ ASAT—aspartate amynotrasferase; ^#^ ALAT—alanine aminotransferase; ^§^ GGT—gamma glytamyl transpeptidase; ^₽^ aPTT, s—activated partial thromboplastin time, seconds; ^Ω^ INR—international normalized ratio.

**Table 2:** Viral infection markers before initiation of antiviral therapy.

Viral Infection Markers	Result	Reference Range
HBs-Ag ^╧^	2200 IU/mL	0.005–150 IU/mL
HBe-Ag ^§^	negative	
Anti-HBe Ab	positive	
Anti-HDV Ab ^¥^ (total)	negative	
Anti-HCV Ab ^Ω^ (total)	negative	
HIV1/2 ^≠^	negative	
HBV DNA ^‡^	1,191,000 copies/mL	not detected

Legend: ^╧^ HBs-Ag—hepatitis B virus surface antigen; ^§^ HBe-Ag—hepatitis B virus excretory antigen; **^¥^** Anti-HDV Ab—hepatitis D virus antibodies, total; ^Ω^ Anti-HCV Ab—hepatitis D virus antibodies, total; **^≠^** HIV1/2—human immunodeficiency virus type 1 and type 2 antibody test; **^‡^** HBV DNA—copies of deoxyribonucleic acid of hepatitis B virus in plasma.

## Data Availability

No new data were created for this case report. All data were included in this article.
